# Unravelling the Morphological, Physiological, and Phytochemical Responses in *Centella asiatica* L. Urban to Incremental Salinity Stress

**DOI:** 10.3390/life13010061

**Published:** 2022-12-25

**Authors:** Hai Ly Hoang, Hafeezur Rehman

**Affiliations:** 1 Faculty of Agronomy, Hue University of Agriculture and Forestry, Hue City 49000, Vietnam; 2 Department of Agronomy, University of Agriculture, Faisalabad 38040, Pakistan

**Keywords:** total flavonoid, phenolics, antioxidant activity, centella, Na^+^ content

## Abstract

*Centella asiatica* L. as a traditional medicinal plant is popular in several Asian countries and characterized by the presence of phytochemicals, such as phenolics and flavonoids. Soil salinity can affect the growth and phytochemical composition in this plant species. In this study, the effects of incremental soil salinity (0, 25, 50, 75, and 100 mM NaCl) on growth, physiological characteristics, total phenolic and total flavonoid contents, including the antioxidant activity of *Centella asiatica* L., were evaluated under greenhouse conditions. Salinity stress reduced growth, biomass production, and total chlorophyll contents, while increasing electrolyte leakage, Na^+^ and Cl^−^ contents in the shoots and roots. With the increase of salt concentration, total phenolic, total flavonoid content and antioxidant activities were increased. The results showed that centella can tolerate saline conditions up to 100 mM NaCl. Na^+^ exclusion from the roots, and that increases of phytochemical content in the shoots were related to the salt tolerance of this species.

## 1. Introduction

*Centella asiatica* L. Urban (centella) as a medicinal plant of the Apiaceae family that has been used to treat a number of diseases, including varicose veins, certain eczemas, hypertonic scars, and keloids [1]. Centella is also considered a valuable plant in the cosmetics and pharmaceutical industries. As a leafy vegetable, this species is consumed as a juice blend in many Asian countries including Vietnam and Malaysia. The medicinal properties of centella are determined by a variety of phytochemicals such as phenolics, flavonoids, and terpenes [2]. The concentration of these compounds is influenced by several environmental stress factors, including salinity [3].

Salinity as an environmental stress factor affects arable lands worldwide, causing an annual monetary loss of approximately $27.3 billion to the agricultural industry [4]. Excessive salt concentration reduces the water potential, resulting in osmotic stress and an increase in the toxic accumulation of sodium and chloride in plant cells. Ionic toxicity and osmotic stress disrupt photosynthetic functions and reduce growth of plants [5]. This results in the accumulation of reactive oxygen species (ROS), including hydrogen peroxide (H_2_O_2_), superoxide anions (O2•−), singlet oxygen (^1^O_2_), and hydroxyl radicals (OH•), resulting in protein, DNA, and lipid damages [6]. The extent of the damage depends on the type, variety, and growth stage of the plant [7].

Plants have evolved antioxidant defense systems to reduce oxidative damage from salinity stress. Phenolic compounds play a major role in scavenging free radicals [8] by acting as hydrogen or electron donors that stabilize and delocalize unpaired electrons or chelate metal ions, preventing the generation of ROS [9]. Plants with higher antioxidant levels have an increased tolerance to damages by ROS [10]. Recent studies have shown various changes in antioxidant compounds when plants are subjected to salinity. Salinity increased the total phenolic content in *Thymus vulgaris* L. [11] and *Brassica oleracea var. acephala* [12]. However, higher salt levels reduced the phenolic content of *Ocimum basilicum* L. [13] and *Nigella sativa* [14]. The significant correlation among phenolic content, antioxidant activity, and salt tolerance is well documented in literature [15]. For example, Sarker et al. [16] reported that salinity stress enhanced total phenolic, total flavonoid, and antioxidant activity of amaranth. Razieh et al. [17] also observed that phenolic content and antioxidant activity were significantly increased by salt stress in wheat. Santander et al. [18] determined that the greatest total phenolic content and antioxidant activity in lettuce prevailed at 50 mM NaCl treatment.

Despite numerous studies reporting the responses of plants to saline stress, there is limited research available on responses of centella to incremental salinity. The present study, therefore, determined the growth and biomass production of centella under saline conditions and their effects on ionic uptake, phytochemical content, and antioxidant activity.

## 2. Materials and Methods

### 2.1. Plant Materials and Experimental Design

Seeds of *Centella asiatica* L. were collected from the Lucky Seed Company, Vietnam. The seeds were sown in trays containing a mixture of coconut fiber and sand. At the third leaf stage, seedlings were transplanted into garden soil-filled, plastic pots (20 × 18 × 10 cm) containing 2.5 kg soil. The soil had a pH of 5.5, 1.3 ECe, 35% organic matter, 0.5 mg L^−1^ Na^+^, 0.88 mg L^−1^ Cl^−^, 0.65% N, 0.71% K_2_O, and 0.62% P_2_O_5_. The study was conducted in a greenhouse in Huong Tra Town, Thua Thien Hue Province, Vietnam, from February to November 2021.

The NaCl was applied as: 0, 25, 50, 75 and 100 mM. The experiment design was a completely randomized block (CRB) design with three replicates, and each replicate included 15 plants. One seedling was transplanted per pot. The soil was drenched with 25, 50, 75, and 100 mM NaCl after transplanting. NaCl was stepped up in daily increments of 25 mM until reaching the final concentration of each treatment. No additional nutrients or fertilizers were added. The experiment was terminated 45 days after transplantation. The plants were evaluated for plant growth, as well as phytochemical and ionic analyses.

### 2.2. Growth Measurement

Plant phenotypes, including the number of leaves, rosette diameter, petiole length, total leaf area, and specific leaf area were recorded. The millimeter graph paper method was used to measure total leaf area per plant [19]. At the end of experiment, shoots and roots were sampled, oven-dried at 60 °C for 60 h, and weighted.

### 2.3. Total Chlorophyll Content Measurement

Two hundred mg of fresh centella leaves were chopped and ground into fine powder in 5 mL 60% acetone (*v/v*). The extractant was filtered, and then the diluted acetone was added to make up the 20 mL final volume. The supernatant was recorded spectrophotometrically at 663 and 645 nm, and the formula given by Lichtenthaler (1987) was used to calculate the total chlorophyll content:

Total chlorophyll = 7.15A663 + 18.71A645

### 2.4. Electrolyte Leakage (EC) Measurement

The method described by Lutt et al. [20] was used to determine electrolyte leakage. The top 4th leaf was collected and thoroughly rinsed with distilled water to remove contamination. The samples were put into stoppered vials containing 10 mL of distilled water and then incubated at 25 °C on a shaker at 100 rpm for 24 h. After incubation, the electrical conductivity of the bathing solution (EC1) was immediately measured. After this, the same leaf samples were placed in an autoclaved at 120 °C for 20 min, and again, a reading EC2 was measured at room temperature using the portable meter HI993310 (Hanna Instrument Company, Woonsocket, RI, USA). The electrolyte leakage was measured as a ratio of EC1/EC2 and expressed as a percentage.

### 2.5. Determination of Hydrogen Peroxide (H_2_O_2_)

Hydrogen peroxide was determined by using the potassium iodide (KI) method. Three mL leaf extract supernatant was mixed with 0.5 mL trichloroacetic acid (TCA) (0.1%), 0.5 mL potassium phosphate buffer (100 mM), and 2 mL reagent 1 mL KI (1 M KI w/v in fresh double-distilled water). A blank probe was made using trichloroacetic acid (0.1%) in the absence of leaf extract. The reaction was developed for 1 h in darkness and absorbance measured at 390 nm. A standard curve was used to estimate the amount of hydrogen peroxide.

### 2.6. Estimation of Ionic Content

After harvesting, nine plants per treatment were separated into aboveground parts and roots. They were washed with de-ionized water, dried at 80 °C in 48 h, and stored at room temperature for further processing. The Na^+^ and Cl^−^ in roots and shoots were determined by using the method described by AcostaMotos et al. [21].

### 2.7. Determination of Total Phenolic Content

Total phenolic content was determined following Velioglu et al. [22]. Plant extracted solution (0.5 mL) was added to diluted Folin–Ciocalteu reagent (2 N, 5 mL), and then 4 mL of 1 M Na_2_CO_3_ and 1 mL water were added to the mixture. The leaf extracts were left to stand for 90 min at 37 °C, and then the phenolic content was determined by using colorimetry at 765 nm. The results were expressed as gallic acid equivalents per milligram (mg GAE g^−1^ DW). The gallic acid solutions were prepared in methanol: water (50:50, *v/v*) as 0, 50, 100, 150, 200, and 250 mg mL^−1^ for standard curve (R^2^ = 0.99).

### 2.8. Determination of Total Flavonoid Content

The flavonoid content was quantified following the method of Zhishen et al. [23]. The 0.5 mL of plant extract solution was added to 1.0 mL methanol, 0.5 mL of aluminum chloride, and 0.5 mL of 1 M potassium acetate and allowed to stand for 30 min. The absorbance of the reaction mixture was detected at 415 nm with a UV/Vis spectrophotometer (Shimazdu UV-2600, Kyoto, Japan). The total flavonoid content was calculated as quercetin from a calibration curve prepared by using quercetin solutions of different concentrations from 12.5 to 100 mg mL^−1^ in methanol.

### 2.9. Determination of Total Antioxidant Activity

The 1,1-Diphenyl -2-picryl-hydrazyl (DPPH) radical degradation method was used to estimate antioxidant activity [24]. The plant extracts (1 mL) were added at different concentrations with volumes equal to the methanolic solution of 10 mL DPPH (100 μM) in a test tube. The mixture was shaken vigorously and was then allowed to stand in the dark. After 15 min, the absorbance was detected at 517 nm as a lower IC_50_ value corresponding to its higher antioxidant activity. This measurement was repeated three times. The IC_50_ values indicate the concentration of the sample.

### 2.10. Statistical Analysis

The data was subjected to analysis of variance (ANOVA) using the Statistical Package for the Social Science (SPSS) software version 12. If the F-test was found significant, mean comparison was performed using the least significant difference (LSD) test at 5% level.

## 3. Results

### 3.1. Plant Growth and Biomass Production

Salinity significantly reduced the centella growth at all NaCl concentrations except 25 mM (Table 1). Plant growth was detrimentally reduced at 100 mM NaCl. High salinity level (100 mM) reduced the number of leaves, leaf area, and specific leaf area by 45%, 38.4% and 35%, respectively. Low and moderate salinity levels (25 and 50 mM NaCl) had no significant effect on the rosette diameter and petiole length of centella, while high NaCl concentration (100 mM) reduced these up to 18.9% and 33.6%, respectively.

Plant dry weight also decreased with incremental salinity, except at 25 mM NaCl (Table 2). The decrease in dry weight ranged from 5.9 to 13.9% with highest reduction of 19.7% at 100 mM NaCl compared with the control.

### 3.2. Total Chlorophyll Content

**Figure 1:**
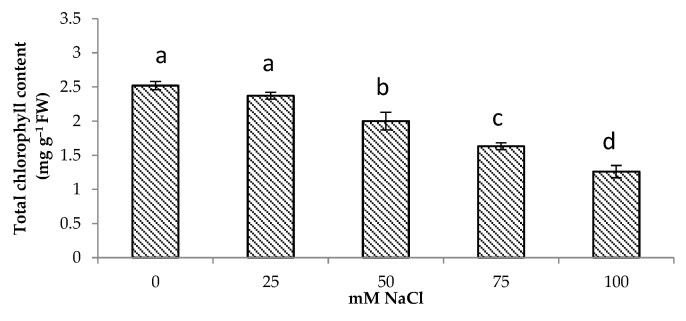
Total chlorophyll content of *Centella asiatica* L. as affected by different salinity levels. The experiment was carried out in triplicate. Different letters represent significant differences at the *p* < 0.05 (FW: fresh weight).

A decrease in the photosynthetic pigment content was observed in centella under salt stress in this study (Figure 1). The total chlorophyll content decreased by 50% at 100 mM NaCl. The highest total chlorophyll content was observed under no salinity followed by of the plants grown under 25 and 50 mM NaCl.

### 3.3. Electrolyte Leakage

**Figure 2:**
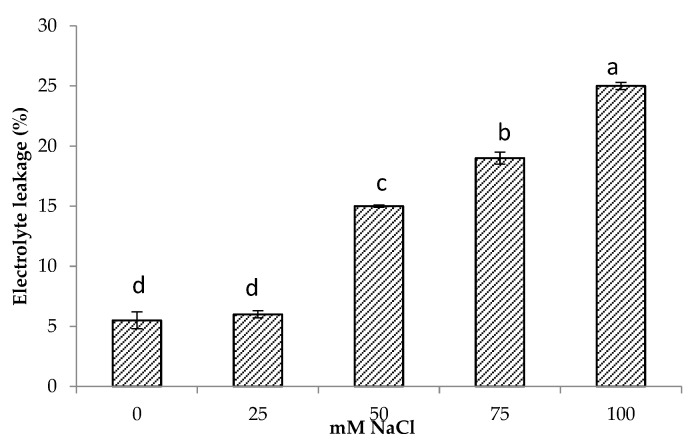
Electrolyte leakage of *Centella asiatica* L. as affected by different salinity levels. The experiment was carried out in triplicate. Different letters represent significant differences at the *p* < 0.05.

The results showed that electrolyte leakage increased with increasing salt concentrations. Minimum electrolyte leakage was found in control plants followed by 25 mM NaCl salinity. Increasing salinity by 50 and 75 mM NaCl and increased electrolyte leakage by 2.7 and 3.4 times compared to control, respectively, while the highest increase of 4.5 times in electrolyte leakage was found at 100 mM NaCl (Figure 2). There was also a significant and positive relationship of electrolyte leakage with shoot Na^+^ (r = 0.85, *p* < 0.001) and root Na^+^ content (r = 0.62, *p* < 0.001) (Table 3).

### 3.4. Hydrogen Peroxide (H_2_O_2_)

**Figure 3:**
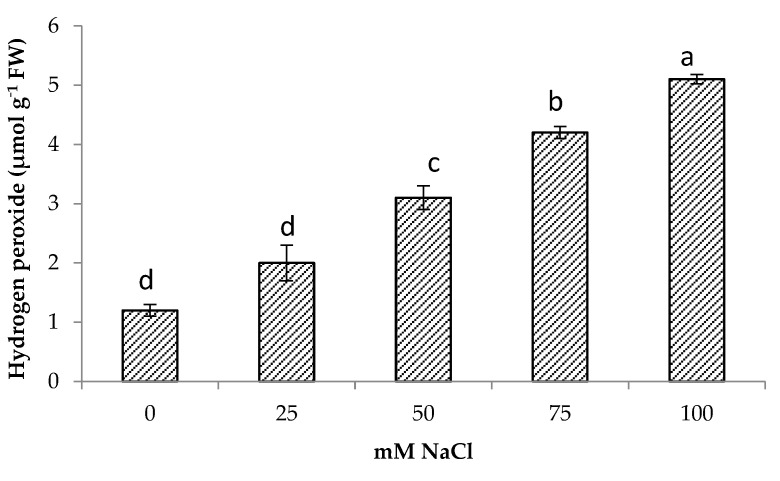
Hydrogen peroxide of *Centella asiatica* L. as affected by different salinity levels. The experiment was carried out in triplicate. Different letters represent significant differences at the *p* < 0.05.

Salt stress increased hydrogen peroxide content significantly in centella. The highest hydrogen peroxide was obtained at 100 mM NaCl (5.1 μmol g^−1^ FW) followed by 75 mM NaCl. The lowest hydrogen peroxide content was found in the control and were comparable to 25 mM NaCl content. At 50 mM NaCl, the hydrogen peroxide in the centella leaf was 4.1 μmol g^−1^ FW (Figure 3). There was also a significant relationship between H_2_O_2_ content and Na^+^ content (Table 3).

### 3.5. Shoot and Root Na^+^ and Cl^−^ Content

**Figure 4:**
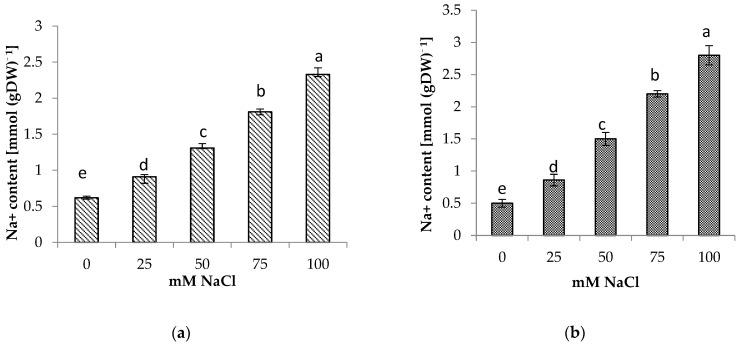
(**a**) Shoot and (**b**) root Na^+^ content of *Centella asiatica* L. as affected by different salinity levels. The experiment was carried out in triplicate. Different letters represent significant differences at the *p* < 0.05.

**Figure 5:**
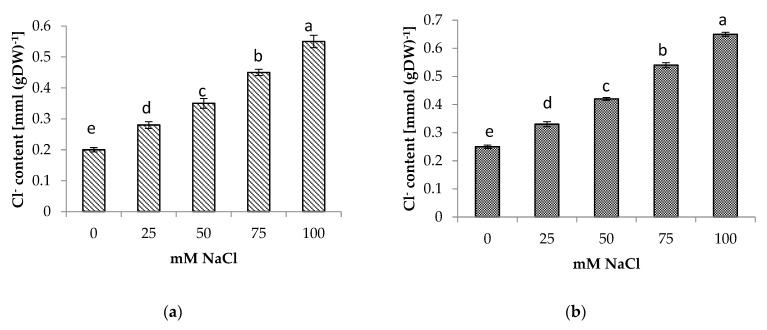
(**a**) Shoot and (**b**) root Cl^−^ content of *Centella asiatica* L. as affected by different salinity levels. The experiment was carried out in triplicate. Different letters represent significant differences at the *p* < 0.05.

The presence of NaCl in the soil medium resulted in the accumulation of Na^+^ and Cl^−^ in the roots and shoots of centella, with higher accumulation found in the roots than in the shoots (Figure 4 and Figure 5). Incremental salinity increased the accumulation of highest Na^+^ content with the highest observed in the roots at 100 mM NaCl, which was 5.7 times greater, followed by 3 and 4.4 times at 50 mM and 75 mM NaCl salinity, respectively. A similar trend in Na^+^ content was observed in the shoots. The highest Na^+^ in the shoot 3.8 times higher was found at 100 mM NaCl than the control.

### 3.6. Phytochemical Content

**Figure 6:**
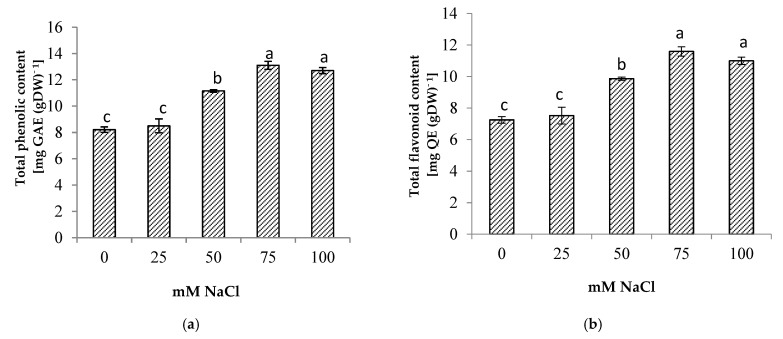
(**a**) Total phenolic, (**b**) total flavonoid (**b**) of *Centella asiatica* L. as affected by different salinity levels. The experiment was carried out in triplicate. Different letters represent significant differences at the *p* < 0.05. (GAE: gallic acid; QE: quercetin; DW: dry weight).

**Figure 7:**
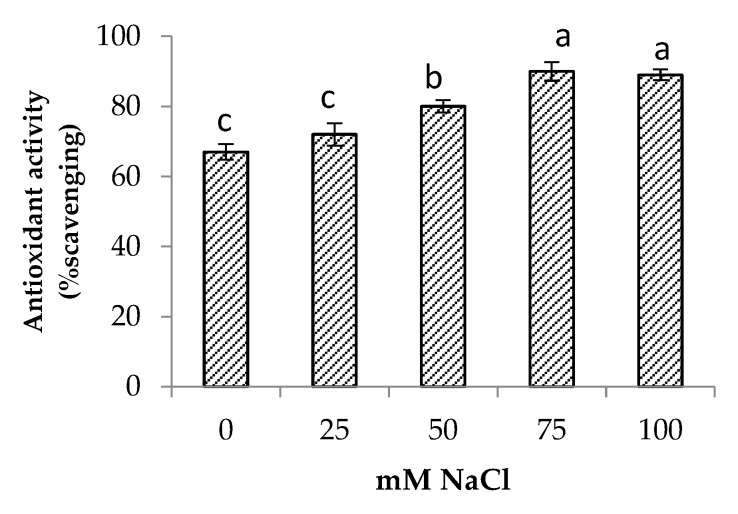
Antioxidant activity of *Centella asiatica* L. as affected by different salinity levels. The experiment was carried out in triplicate. Different letters represent significant differences at the *p* < 0.05.

Salinity stress significantly increased the total phenolic and total flavonoid contents of the centella (Figure 6). The highest total phenolic and flavonoid contents were found in the plants at 75 mM NaCl. The increase in total phenolic and total flavonoid contents augmented the antioxidant activity up to 34% when compared to the control (Figure 7). At a high salt concentration (100 mM NaCl) their accumulation was reduced; however, it was not significantly different from 75 mM NaCl salinity.

## 4. Discussion

The results of the present study showed that centella growth was decreased by incremental salinity. A similar response in plant growth was reported in *Portulaca oleracea* L. [25], *Prosopis strombulifera* [26], and *Tetragonia decumbens* [27] due to salt stress. High saline concentrations reduced growth by decreasing the uptake of water and nutrients by the plants [28], accumulating toxic ions in the plant cells, and disrupting the metabolic pathways [29]. In this study, specific leaf area decreased with an increase in salinity. Burslem et al. [30] showed that a higher leaf thickness is associated with an increase in the ratio of mesophyll area available for the absorption of CO_2_ per unit leaf area, thereby enhancing CO_2_ assimilation and biomass production. However, Omami et al. [31] found that CO_2_ assimilation decreased with increasing salinity in amaranth. They suggested that the lower specific leaf area in salt stressed plants overloaded the leaves with inorganic and organic solutes, thereby permitting osmotic flow but limiting the efficient use of carbon. Increase in the leaf thickness could be an adaptation of the plant to increase intercellular space and to counteract the decrease of transpiration [32].

The current study indicated that the root biomass decreased under high salt concentration treatments. According to Banaka et al. [33], the main reasons for reduced plant growth and biomass under high salinity were ion toxicity and nutrient imbalance. Moreover, the increase of soluble salts in the soil leads to an increase of osmotic pressure and a reduction of water potential, thus reducing the water uptake by the root [34]. In this study, although salt stress inhibited plant growth and decreased biomass production, the root/shoot dry weight increased. This indicated that salinity affected the aboveground part more severely than the underground part and the plant had the ability to change biomass allocation. It means that the plants had the ability to maintain the root system while salt stress inhibited shoot growth. This response is one of the most popular strategies of plants to adapt to abiotic stress.

Chlorophyll content is an important factor in assessing photosynthetic activity in plants [35]. The results showed a decrease in the total chlorophyll content of the centella under saline conditions. Previous studies showed that the depletion of photosynthetic pigments reducing plant growth and crop yield under saline stress was also evident from a significant relationship between total chlorophyll content and biomass production in the present study (r = 0.9, *p* < 0.001) (Table 3). This was observed in *Amaranthus tricolor* [36], *Typha domingensis* [37], and *Lactuca sativa* L. [38].

There was also a negative correlation between the total chlorophyll content and the shoot Na^+^ content (r = −0.67, *p* < 0.001), showing degradation of photosynthetic pigments under the incremental salinity (Table 3). This leads to a reduction in biomass production as indicated by the negative correlation between fresh weight/dry weight with Na^+^ concentration (Table 3). Depletion of chlorophyll under saline conditions may be caused by the accumulation of toxic ions, such as Na^+^ and Cl^−^ inhibiting the enzymes function responsible for chlorophyll synthesis [39]. Zahra et al. [40] also reported that salt stress could reduce the CO_2_ supplement through hydrostatic stomata closure or by changing the mesophyll conductance. According to Farhat et al. [41], a high salt concentration may damage the thylakoid membranes and protein modulation by inhibiting photosynthesis. Recent studies showed that the formation of ROS disrupted the chloroplasts and ultimately reduced the total population of *Brassica napus* [42], *Chenopodium quinoa* [43] and *Solanum lycopersicum* [44].

In this study, the centella was able to maintain membrane stability under slight salt stress (Figure 2) as evident from the electrolyte leakage which increased when the plants were subjected to a high salt concentration. A similar response was observed by ElYacoubi et al. [45] in ryegrass and by Behdad et al. [46] in licorice. This was mainly due to the efflux of K^+^ and the flow of counter ions (Cl^–^, HPO_4_^2–^, NO_3_^–^, citrate^3–^, and malate^2–)^ counterbalancing the efflux of K^+^ [47]. According to Tavakkoli et al. [48], the distribution of Na^+^ within cells and organs may subsequently cause toxic effects on membrane permeability and increased electrolyte leakage.

In this study, the increase of Na^+^ and Cl^−^ concentrations in the tissues was accompanied by salinity stress. High accumulations of Na^+^ and Cl^−^ reduced plant growth. High Na^+^ concentrations interfered with the absorption of K^+^ and Ca^2+^ ions and disturbed stomatal regulation, thereby inhibiting photosynthesis and growth. High Cl^−^ concentrations caused the degradation of chlorophyll, leading to a reduction in the photosynthesis rate [48]. However, plants have different coping mechanisms for dealing with Na^+^ toxicity. Some plants transport Na^+^ from the roots to the leaves where it is retained in the vacuoles, whereas others store Na^+^ in the roots [49]. Salt tolerance is associated with the ability to limit the uptake and/or to transport Na^+^ from the root zone to aerial parts [50]. Based on the distribution of Na^+^ and Cl^−^ between shoots and roots, a similar mechanism could occur in centella. The accumulation of Na^+^ and Cl^−^ in the roots provided a mechanism for centella to cope with salinity in the rooting medium. This mechanism reduced the transport of Na^+^ and Cl^−^ to the leaves, thereby reducing the impact of the toxic ions to the aboveground parts of the plant. The leaves of centella are usually harvested, which is advantageous for growing this plant in saline environments. This mechanism has also been reported in amaranth [36] and rapeseed [51].

One of the effects of salt stress on plants is the overproduction of ROS, which leads to oxidative stress. However, plants have evolved mechanisms to counteract the effects of this process by producing compatible metabolites and different antioxidants [10]. Phenolic compounds are the most abundant secondary metabolites in the plant kingdom which have a pivotal effect in scavenging the excessive ROS. Flavonoids as a group belong to phenolic compounds and are known to have antioxidant properties [10]. The presence of phenolics and flavonoids in plants contributed to the prevention of cell damage by abiotic stress, as demonstrated by several studies on peas [52] and kale [53]. These compounds neutralize the radicals accumulated in lipids or prevent their breakdown into free radicals. Furthermore, they can inhibit lipoxygenase activity, thus preventing lipid peroxidation [54,55]. The result showed that there was a significant increase in the phenolic and flavonoid content in response to salt stress. The increase in phenolic and flavonoid content indicates that they play a significant role in the adaptation of centella to salinity as evident from a positive correlation between total phenolic content and antioxidant activity in the present study (Table 3). The increase in these compounds is related to their function as a non-enzyme antioxidant to counteract the increase of ROS and hence contribute to the plant’s health under salt stress. In the present study, antioxidant activity of the centella leaf increased with the salt treatments, and the highest antioxidant activity was observed at 100 mM NaCl. This finding is consistent with the important relationship that exists between antioxidant activity and the total phenolic content in the leaves of *Leucojum aestivum* and *Lactuca sativa* under salt stress conditions [19,56]. Although the centella was also negatively affected by salt stress, as demonstrated by yield decline and increased accumulation of Na^+^ and Cl^−^ ions, the study results showed an increase in phytochemicals content and antioxidant activity in centellas. This opens the way to cultivating this plant in saline soils to boost the production of bioactive compounds used in the pharmaceutical and cosmetics industries. However, studies on extraction techniques for specific bioactive compounds should be carried out to ensure the exclusion of ions and impurities.

## 5. Conclusions

Salinity stress caused a reduction in biomass yield and induced some physiological and phytochemical modification in centella. The results indicated that *Centella asiatica* showed moderate tolerance to severe salt stress, which was attributed to the exclusion of Na^+^ and Cl^−^ in the root to protect the aboveground plant tissues from salt toxicity and to increase the total phenolic and flavonoid content of the centella. The centella is an herb with a rich source of phytochemical content. Thus, the response of the centella under salt conditions may be used to improve the production of bioactive compounds to be used in the manufacture of pharmaceuticals, supplements, food, and cosmetics.

## Figures and Tables

**Table 1:** Morphological parameters of *Centella asiatica* L. as affected by different salinity levels. The experiment was carried out in triplicate. The data are presented as treatment mean ± SD. Different letters in the same column represent significant differences at the *p* < 0.05.

Treatments (mM NaCl)	Number of Leaves	Leaf Area (cm^2^)	Specific Leaf Area (cm^2^ g^−1^)	Rosette Diameter (cm)	Petiole Length (g)
0	20.50± 5.20 ^a^	24.20 ± 2.61 ^a^	347.50 ± 54.61 ^a^	17.30 ± 2.53 ^a^	6.06 ± 1.12 ^a^
25	21.62 a ± 6.12 ^a^	26.00 ± 3.22 ^a^	358.20 ± 93.41 ^a^	18.12 ± 3.34 ^a^	6.37 ± 2.11 ^a^
50	18.40 b ± 5.50 ^b^	22.21 ± 5.81 ^b^	312.80 ± 32.63 ^b^	17.95 ± 2.81 ^a^	5.80 ± 0.92 ^a^
75	15.60 c ± 7.01 ^c^	17.83 ± 3.53 ^c^	265.20 ± 49.74 ^c^	15.53 ± 4.21 ^b^	5.11 ± 1.41 ^b^
100	11.21 d ± 3.52 ^d^	14.92 ± 5.11 ^d^	225.40 ± 77.52 ^d^	14.03 ± 3.14 ^b^	4.02 ± 0.84 ^b^

**Table 2:** Biomass production of *Centella asiatica* L. as affected by different salinity levels. The experiment was carried out in triplicate. The data are presented as treatment mean ± SD. Different letters in the same column represent significant differences at the *p* < 0.05.

Treatments	Fresh Weight Leaf (g/plant)	Dry Weight Leaf (g/plant)	Fresh Weight Root (g/plant)	Dry Weight Root (g/plant)	Root/Shoot Ratio Dry Weight (g)
(mM NaCl)					
0	50.33 ± 5.21 ^c^	10.06 ± 2.15 ^c^	37.55 ± 6.11 ^a^	7.51 ± 0.85 ^a^	0.40 ± 0.03 ^a^
25	52.65 ± 3.57 ^c^	10.53 ± 3.21 ^c^	33.25 ± 5.40 ^b^	6.65 ± 0.72 ^b^	0.42 ± 0.07 ^a^
50	61.00 ± 7.54 ^a^	12.20 ± 2.20 ^a^	29.65 ± 6.41 ^c^	5.93 ± 0.63 ^c^	0.49 ± 0.04 ^a^
75	57.15 ± 3.90 ^b^	11.42 ± 1.50 ^b^	22.25 ± 8.01 ^d^	4.45 ± 0.51 ^d^	0.58 ± 0.02 ^b^
100	46.71 ± 6.52 ^d^	9.34 ± 1.15 ^d^	20.25 ± 7.24 ^d^	4.05 ± 0.82 ^d^	0.80 ± 0.09 ^a^

**Table 3:** Correlation coefficients among some morphological and physiological characteristics.

	Fresh Weight	Dry Weight	Total Chlorophyll Content	Electrolyte Leakage	Hydrogen Peroxide	Shoot Na^+^ Content	Root Na^+^ Content	Shoot Cl^−^ Content	Root Cl^−^ Content	Total Phenolic Content	Total Flavonoid Content	Antioxidant Activity
Fresh weight	1											
Dry weight	0.90 **	1										
Total chlorophyll content	0.85 **	0.83 **	1									
Electrolyte leakage	−0.67 **	−0.70 **	−0.67 **	1								
Hydrogen peroxide	−0.56 **	−0.59 **	−0.58 **	0.81 **	1							
Shoot Na^+^ content	−0.67 **	−0.62 **	−0.59 **	0.85 **	0.80 **	1						
Root Na^+^ content	−0.56 **	−0.58 **	−0.53 **	0.62 **	0.65 **	0.61 **	1					
Shoot Cl^−^ content	−0.68 **	−0.65 **	−0.60 **	0.76 **	0.74 **	0.67 **	0.55 **	1				
Root Cl^−^ content	−0.53 **	−0.55 **	−0.52 **	0.60 **	0.53 **	0.45 **	0.61 **	0.55 **	1			
Total phenolic content	−0.54 **	−0.67 **	−0.51 **	0.31 ^ns^	0.23 ^ns^	0.65 **	0.52 **	0.50 **	0.52 **	1		
Total flavonoid content	−0.70 **	−0.64 **	−0.54 **	0.75 **	0.62 **	0.83 **	0.61 **	0.65 **	0.67 **	0.84 **	1	
Antioxidant activity	−0.20 ^ns^	−0.31 ^ns^	−0.03 ^ns^	−0.16 ^ns^	−0.21 ^ns^	0.56 **	0.60 **	0.58 **	0.60 **	−0.78 **	−0.56 *	1

* and **: significant difference at 5 and 1%, respectively; ns: not significant.

## Data Availability

Data recorded in the current study are available in all tables and figures of the manuscript.
